# Supplementary material for: IGF-1 and IGFBP-1 as Possible Predictors of Response to Lifestyle Intervention—Results from Randomized Controlled Trials
Source: Int J Mol Sci. 2024 Jun 10;25(12):6400. doi: 10.3390/ijms25126400 (PMC11203659; doi:10.3390/ijms25126400)
Supplement: Supplementary file 1 [file ijms-25-06400-s001.zip › ijms-3009414-supplementary.pdf]

Supplemental Material

IGF-1 and IGFBP-1 as Possible Predictors of Response to Lifestyle Intervention—Results from Randomized Controlled Trials

Nina M. T. Meyer [1,2,3], Stefan Kabisch [1,2,3], Ulrike Dambeck [3], Caroline Honsek [3], Margrit Kemper [2,3], Christiana Gerbracht [3], Ayman M. Arafat [1], Andreas L. Birkenfeld [2,4,5,6], Peter E. H. Schwarz [2,7,8], Jürgen Machann [2,4,9], Martin A. Osterhoff [3], Martin O. Weickert [10,11,12,13] and Andreas F. H. Pfeiffer [1,2,3,*]

[1] Department of Endocrinology and Metabolism (Diabetes and Nutritional Medicine), Charité Universitätsmedizin Berlin, 10117 Berlin, Germany; nina.meyer@charite.de (N.M.T.M.)
[2] German Center for Diabetes Research (DZD), 85764 München-Neuherberg, Germany
[3] Department of Clinical Nutrition/DZD, German Institute of Human Nutrition Potsdam-Rehbruecke, 14558 Nuthetal, Germany
[4] Institute of Diabetes Research and Metabolic Diseases (IDM), Helmholtz Center Munich, Eberhard Karls University of Tübingen, 72076 Tübingen, Germany
[5] Department of Internal Medicine IV—Endocrinology, Diabetology, and Nephrology, University Hospital Tübingen, 72076 Tübingen, Germany
[6] Department of Diabetes, School of Life Course Science and Medicine, King's College London, London WC2R 2LS, UK
[7] Department for Prevention and Care of Diabetes, Clinic of Medicine III, Faculty of Medicine Carl Gustav Carus, Technische Universität Dresden, 01307 Dresden, Germany
[8] Paul Langerhans Institute Dresden, Helmholtz Center Munich, Faculty of Medicine, Technische Universität Dresden, 01307 Dresden, Germany
[9] Section on Experimental Radiology, Department of Diagnostic and Interventional Radiology, University Hospital Tübingen, 72076 Tübingen, Germany
[10] Warwickshire Institute for the Study of Diabetes, Endocrinology and Metabolism, University Hospitals Coventry and Warwickshire NHS Trust, Coventry CV2 2DX, UK
[11] The ARDEN NET Centre, ENETS CoE, University Hospitals Coventry and Warwickshire NHS Trust, Coventry CV2 2DX, UK
[12] Centre of Applied Biological & Exercise Sciences (ABES), Faculty of Health & Life Sciences, Coventry University, Coventry CV1 5FB, UK
[13] Translational & Experimental Medicine, Division of Biomedical Sciences, Warwick Medical School, University of Warwick, Coventry CV4 7AL, UK
* Correspondence: andreas.pfeiffer@charite.de; Tel.: +49-30-450-514-422

Table of Contents

1. Supplemental Table S1 a) and b) p. 2
2. Supplemental Table S2 a) and b) p. 4

Supplemental Table S1 a) and b). Differences of baseline values between subgroups of a) IGF-1 baseline levels and b) IGFBP-1 baseline levels.

Parameters	Baseline value	n	Baseline value	n	P for between group differences
		(a)			
	**IGF-1 < 134.2 µg/L**		**IGF-1 ≥ 134.2 µg/L**		
IGF-1 [µg/L]	99.9 ± 23.3	172	183.5 ± 41.5	173	**<0.001**
IGFBP-1 [µg/L]	2.2 [1.2; 4.4]	172	2.1 [0.9; 3.7]	173	**0.036** [a]
IGFBP-2 [µg/L]	269.6 [148.1; 453.6]	172	251.5 [133.9; 385.2]	173	0.304
Body Mass Index [kg/m²]	30.8 ± 5.2	172	31.1 ± 5.6	173	0.596
Waist-to-hip ratio [cm/cm]	0.94 ± 0.09	171	0.93 ± 0.09	170	0.444
Body fat content$_{\text{-BIA}}$ [%]	35.1 ± 8.6	153	34.2 ± 8.5	159	0.333
Visceral fat volume$_{\text{-MRI}}$ [l]	5.7± 2.5	124	5.4 ± 2.3	101	0.394
Intrahepatic Lipid Content$_{\text{-MRS}}$ [%-abs.]	7.0 [3.0; 14.7]	127	7.2 [3.0; 14.2]	104	0.798 [a]
Fasting glucose [mmol/L]	5.8 ± 0.7	172	5.7 ± 0.7	173	0.174
2-h glucose [mmol/L]	8.3 ± 1.5	172	8.1 ± 1.6	173	0.169
Fasting insulin [pmol/L]	79.7 [55.8; 108.2]	171	66.0 [49.6; 99.7]	166	**0.048** [a]
HOMA-IR	3.0 [1.9; 3.9]	171	2.4 [1.6; 3.7]	166	**0.034** [a]
Matsuda Index	2.4 [1.6; 3.3]	124	2.8 [1.9; 3.6]	114	0.056 [a]
HIRI	37.5 [31.3; 45.4]	128	36.7 [30.0; 42.6]	114	0.213
IGI	11.7 [7.5; 21.4]	128	11.6 [7.5; 19.2]	114	0.938
DI	28.2 [19.5; 43.5]	124	33.6 [23.1; 45.4]	114	0.076
		(b)			
	**IGFBP-1 < 2.13 µg/L**		**IGFBP-1 ≥ 2.13 µg/L**		
IGF-1 [µg/L]	141.5 ± 48.5	172	142.1 ± 58.5	173	.920

IGFBP-1 [µg/L]	1.0 [0.7; 1.5]	172	4.1 [2.8; 6.8]	173	**<.001** [a]
IGFBP-2 [µg/L]	223.6 [119.5; 369.2]	172	310.2 [175.4; 463.2]	173	**<.001** [a]
Body Mass Index [kg/m²]	31.8 ± 5.0	172	30.0 ± 5.7	173	**.002**
Waist-to-hip ratio [cm/cm]	0.94 ± 0.08	170	0.93 ± 0.10	171	.317
Body fat content-BIA [%]	35.5 ± 8.0	153	33.8 ± 9.0	159	.077
Visceral fat volume-MRI [l]	5.9 ± 2.1	121	5.1 ± 2.7	104	**.014**
Intrahepatic Lipid Content -MRS [%-abs.]	9.4 [5.1; 17.1]	126	4.1 [1.5; 9.2]	105	**<.001** [a]
Fasting glucose [mmol/L]	5.8 ± 0.6	172	5.7 ± 0.7	173	.136
2-h glucose [mmol/L]	8.2 ± 1.5	172	8.3 ± 1.6	173	.406
Fasting insulin [pmol/L]	82.0 [59.3; 115.3]	165	64.2 [43.2 98.0]	172	**<.001** [a]
HOMA-IR	3.0 [2.1; 4.1]	165	2.3 [1.5; 3.4]	172	**<.001** [a]
Matsuda Index	2.4 [1.7; 3.2]	131	2.9 [2.2; 4.6]	107	**<.001** [a]
HIRI	38.3 [32.8; 46.6]	134	34.9 [28.0; 40.9]	108	**<.001** [a]
IGI	13.7 [8.9; 24.2]	134	8.5 [5.7; 15.7]	108	**<.001** [a]
DI	32.9 [22.1; 46.1]	131	28.4 [19.5; 39.2]	107	.100

Between-group differences of normally distributed variables were tested via Student's t-Test (two-tailed) and of non-normally distributed parameters via Mann-Whitney-U Test. a non-parametric testing. Significant p-values are bolded. Abbreviations: IGF-1 Growth Factor 1. Insulin-like IGFBP1/-2: Insulin-like Growth Factor Binding Protein-1/-2. BIA Bioelectrical impedance analysis. MRI Magnetic resonance imaging. MRS Magnetic resonance spectroscopy. abs absolute. HOMA Homeostatic model assessment. IR Insulin Resistance. HIRI Hepatic insulin resistance index (Abdul-Ghani). IGI Insulinogenic Index (Seltzer). DI Disposition Index-2.

Supplemental Table S2 a) and b). Changes of metabolic parameters over time in association with a) IGF-1 baseline levels, comparing highest vs. lowest tertile and b) IGFBP-1 baseline levels, comparing lowest vs. highest tertile.

**Parameters**	**Mean Difference**	**95% CI**		**p**	**d / r**
		Lower	Upper		
			(a)		
	**Subgroups of IGF-1 baseline levels: third vs first tertile**				
Δ IGF-1 [μg/L]	-42.67	-54.20	-31.14	**<.001**	-.96
Δ IGFBP-1[μg/L]	-0.06	-0.83	0.71	.695 [a]	-.03
Δ IGFBP-2 [μg/L]	29.19	-18.93	77.32	.541 [a]	-.03
Δ Body Mass Index [kg/m²]	-0.34	-0.81	0.12	.074	-.19
Δ Waist-to-hip ratio [cm/cm]	0.01	-0.01	0.03	.075	.19
Δ Body fat content-BIA [%]	0.31	-0.80	1.42	.291	.08
Δ Visceral fat volume-MRI [l]	-0.24	-0.52	0.03	**.040**	-.31
Δ Intrahepatic Lipid Content -MRS [%-abs.]	-1.45	-3.27	0.37	**.019** [a]	-.20
Δ Fasting glucose [mmol/L]	0.03	-0.13	0.18	.376	.04
Δ 2-h glucose [mmol/L]	-0.29	-0.77	0.19	.119	-.16
Δ Fasting insulin [pmol/L]	-14.69	-36.90	7.52	.118 [a]	-.10
Δ HOMA-IR	-0.49	-1.29	0.31	.191 [a]	-.09
Δ Matsuda Index	0.73	0.18	1.29	**.004** [a]	-.23
Δ HIRI	-2.35	-4.89	0.20	.075 [a]	-.14
Δ IGI	-4.47	-9.86	0.92	.072 [a]	-.15
Δ DI	-2.35	-16.84	12.15	.777 [a]	-.02

(b)					
**Subgroups of IGFBP-1 baseline levels: first vs. third tertile**					
Δ IGF-1 [μg/L]	13.40	0.92	25.89	**.018**	.28
Δ IGFBP-1 [μg/L]	2.53	1.39	3.67	**<.001**	-.28
Δ IGFBP-2 [μg/L]	12.37	-37.60	62.35	.492	-.05
Δ Body Mass Index [kg/m²]	-0.43	-0.88	0.01	**.027**	-0.26
Δ Waist-to-hip ratio [cm/cm]	0.00	-0.02	0.02	.385	.04
Δ Body fat content-BIA [%]	-0.46	-1.52	0.59	.193	-.12
Δ Visceral fat volume-MRI [l]	-0.12	-0.43	0.18	.213	-.14
Δ Intrahepatic Lipid Content -MRS [%-abs.]	-2.01	-4.14	0.13	**.006**	-.24
Δ Fasting glucose [mmol/L]	-0.04	-0.18	0.10	.303	-.07
Δ 2-h glucose [mmol/L]	-0.27	-0.75	0.20	.128	-.15
Δ Fasting insulin [pmol/L]	-2.60	-15.57	10.37	.356	-.06
Δ HOMA-IR	-0.16	-0.66	0.35	.240	-.08
Δ Matsuda Index	0.83	0.13	1.54	.093 [a]	-.14
Δ HIRI	-2.27	-5.01	0.47	.076	-.14
Δ IGI	-0.05	-8.14	8.03	.203 [a]	-.10
Δ DI	9.44	-10.21	29.09	.826 [a]	-.02

IGF-1: first tertile ≤ 112.6 μg/L, third tertile ≥ 159.1 μg/L. IGFBP-1 first tertile ≤ 1.29 μg/L; IGFBP-1 third tertile ≥ 3.2 μg/L. Between-group differences of normally distributed variables were tested via Welch t-test (one-tailed) and of non-normally distributed parameters via Mann-Whitney-U Test. a non-parametric testing. p for within-group difference, respectively. Significant p-values are bolded. Effect sizes are given as d= Cohen's d for parametric testing, or Pearson's correlation coefficient r for non-parametric testing. Δ=

Delta. Abbreviations: IGF-1 Growth Factor 1. Insulin-like IGFBP1/-2: Insulin-like Growth Factor Binding Protein-1/-2. BIA Bioelectrical impedance analysis. MRI Mag-netic resonance imaging. MRS Magnetic resonance spectroscopy. abs absolute. HOMA Homeostatic model assessment. IR Insulin Resistance. HIRI Hepatic insulin resistance index (Abdul-Ghani). IGI Insulinogenic Index (Seltzer). DI Disposition Index-2.
